# Water, sanitation, and hygiene (WASH) practices in Africa: exploring the effects on public health and sustainable development plans

**DOI:** 10.1186/s41182-024-00614-3

**Published:** 2024-10-09

**Authors:** Olalekan John Okesanya, Gilbert Eshun, Bonaventure Michael Ukoaka, Emery Manirambona, Olaleke Noah Olabode, Ridwan Olamilekan Adesola, Inibehe Ime Okon, Safayet Jamil, Amandeep Singh, Don Eliseo Lucero-Prisno, Habib Mohammad Ali, A. B. M. Alauddin Chowdhury

**Affiliations:** 1 Department of Medical Laboratory Science, Neuropsychiatric Hospital, Aro, Abeokuta, Nigeria; 2 Seventh Day Adventist Hospital, Agona-Asamang, Ghana; 3 Department of Internal Medicine, Asokoro District Hospital, Abuja, Nigeria; 4 Research Unit, Global Health Focus, Gitega, Burundi; 5 Department of Medical Laboratory Science, Obafemi Awolowo University Teaching Hospitals Complex, Ile Ife, Osun State Nigeria; 6 Department of Veterinary Medicine, Faculty of Veterinary Medicine, University of Ibadan, Ibadan, Nigeria; 7 Department of Neurosurgery, Hospital of the Babcock University, Ilisan Remo, Ogun State Nigeria; 8 Department of Public Health, Daffodil International University, Dhaka, 1216 Bangladesh; 9 Department of Pharmaceutics, ISF College of Pharmacy, Moga, Punjab 142001 India; 10 Department of Global Health and Development, London School of Hygiene and Tropical Medicine, London, UK; 11 Department of Media Studies and Journalism, University of Liberal Arts Bangladesh (ULAB), Dhaka, Bangladesh

**Keywords:** Water, sanitation, and hygiene, WASH, Public health, Africa, Sustainable Development Goals

## Abstract

**Background:**

Suboptimal water, sanitation, and hygiene (WASH) practices constitute a serious public health risk, affecting one-third of the world's population. Remarkable progress has been made to improve WASH; however, challenges remain, with rapid population growth adding pressure on WASH systems. This study explores the current state of WASH practices and diseases in Africa, identifies challenges, and proposes public health recommendations for sustainable implementation.

**Main body:**

The staggering burden of WASH-related diseases in low- and middle-income countries (LMICs), particularly in Africa, threatens public health, with millions of deaths and disability-adjusted life years (DALYs) attributed to poor WASH practices annually. Notable challenges plaguing WASH practices in the region include poverty, malnutrition, poor data reporting, illiteracy, climate change, and poor healthcare financing. This results in adverse health consequences, including waterborne infections like cholera, typhoid, dysentery, and diarrheal diseases. Additionally, neglected tropical diseases (NTDs) such as intestinal worms, schistosomiasis, trachoma, lost productivity, and environmental pollution from soil and underground water contamination have been implicated. Geographical disparities, cultural norms, and inadequate funding further complicate efforts to improve WASH infrastructure and practices. Globally concerted efforts are required to address these issues and permit WASH practices to protect human health by preventing infectious diseases and contributing to economic growth. Strong financial frameworks, skills training, and tools like WASH Fit are recommended for a stronger WASH approach in Africa.

**Conclusion:**

The consequences of poor WASH extend beyond public health, impacting economic growth, gender equality, and environmental sustainability. WaterAid’s policy recommendations prioritizing government administration, institutional capacity enhancement, and more financial resources are expedient.

## Introduction

Water, sanitation, and hygiene (WASH) practices are crucial for individual and community health. Access to safe drinking water, proper sanitation facilities, and consistent hygiene habits encompass necessities for mitigating the spread of diseases, improving overall well-being, and strengthening health policy implementation. From regular handwashing to ensuring safe waste disposal, these seemingly simple practices profoundly impact lives worldwide. While comprehensive WASH coverage is critical to enhancing the standard of living, in 2022, an estimated 2.2 billion people globally lacked potable water [1]. Further compounding, more than 260 million people globally travel long distances to access a water source, while about 159 million drink from contaminated water sources [2]. According to the World Health Organization (WHO), about 1.4 million deaths were associated with suboptimal WASH practices in 2019, with approximately 74 million disability-adjusted life years (DALYs) and over 1 million diarrhea-associated deaths in Africa [3]. Open defecation is still widely practiced, with over 673 million people utilizing open-pit latrines and poor sanitation practices [4].

The Sustainable Development Goals of the United Nations General Assembly emphasize the need for global access to safe drinking and potable water. However, sub-Saharan Africa (SSA) bears significant risk, with a twofold rise in population and a limited availability of hygiene and sanitation facilities. This limitation leaves the region grappling with hygiene-related diseases, further highlighting the importance of coordinated global efforts to tackle these public health challenges and ensure the safety of communities worldwide [4]. Worsening indices are noted across African nations compared to other continents, with 24 African countries leading the first-twenty-five highest mortality rate attributed to exposure to unsafe WASH services [3]. The UN-Water Global Analysis and Assessment of Sanitation and Drinking Water (GLASS 2018–2019) showed limited progress in WASH services toward well-managed urban drinking water targets. While some countries demonstrated accelerating improvements, others suffered from poor data reporting [5]. Apart from the above-mentioned, other challenges have been identified. Across the region, over 70% of rural water schemes are inoperable, further compounding the hurdles of assessing potable water and attaining the UN 2030 SDG 6. Concerns, such as persistent rural–urban inequities, where some geographical settlements have access to water and other basic amenities while others wallow in deficit, pose impediments to bridging these public health and development gaps [2, 6].

Recognizing WASH as a major public health concern helps disrupt the intergenerational cycles of malnutrition, in which inadequate food intake and diseases are influenced by variables such as a lack of safe, potable water, sanitation, and hygiene. It is critical to shift from interventions aimed primarily at certain groups, such as under-five children and pregnant or breastfeeding women, to a more holistic strategy that incorporates all members of the community [7]. Africa suffers from unique issues in the area of WASH, making this study crucial. The continent is confronted with enormous obstacles, including growing urbanization, poverty, and poor governance, all of which exacerbate the already substantial burden of diseases associated with WASH. These challenges are made worse by climate change impacts, including water scarcity and extreme weather events. Additional barriers to WASH implementation in Africa are societal attitudes and cultural norms towards hygiene practices [2].

A comprehensive examination of WASH practices among African communities is necessary due to the complexity involved, especially in light of ongoing public health concerns like climate change and outbreaks of avoidable diseases. This is necessary to emphasize hygiene's critical role in reducing the prevalence of infectious and communicable diseases such as polio, cholera, diarrhea, schistosomiasis, typhoid, and other waterborne and hygiene-related illnesses prevalent in many African nations. This study aims to comprehensively analyze WASH conditions in Africa, identify challenges and barriers, and propose public health recommendations for sustainable implementation in Africa.

## WASH-related disease burden in LMICs

WASH-related diseases predominantly impact vulnerable populations, including infants, under-five children, pregnant women, and those in resource-constrained settings. Ensuring adequate WASH is crucial for preventing illnesses and strengthening the healthcare system. It also improves pandemic preparedness and combats antibiotic resistance [8]. Out of 1.50 million diarrheal deaths in 2012, poor water usage resulted in 502,000 deaths, and poor sanitation practices accounted for 280,000 deaths, while the deaths caused by poor water and sanitation amounted to 685,000 deaths [9]. In low- and middle-income countries (LIMCs), the number of deaths in 2016 attributable to poor WASH practices was remarkable. This mortality amounted to over 1.6 million, with approximately 105 million DALYs that would have been preventable if WASH services were adequately implemented across affected regions [10]. It was also responsible for 60% (829,000) of all diarrheal deaths, with 297,000 of these deaths occurring among children under the age of five. WASH-associated acute respiratory infections and malnutrition accounted for 13% (370,370) and 16% (28,194) of deaths for the period, respectively. Other diseases such as schistosomiasis, malaria, soil-transmitted helminth infections, and trachoma also contributed to the overall morbidity and mortality burden for the period, highlighting the serious health consequences of poor WASH practices [10].

**Fig. 1:**
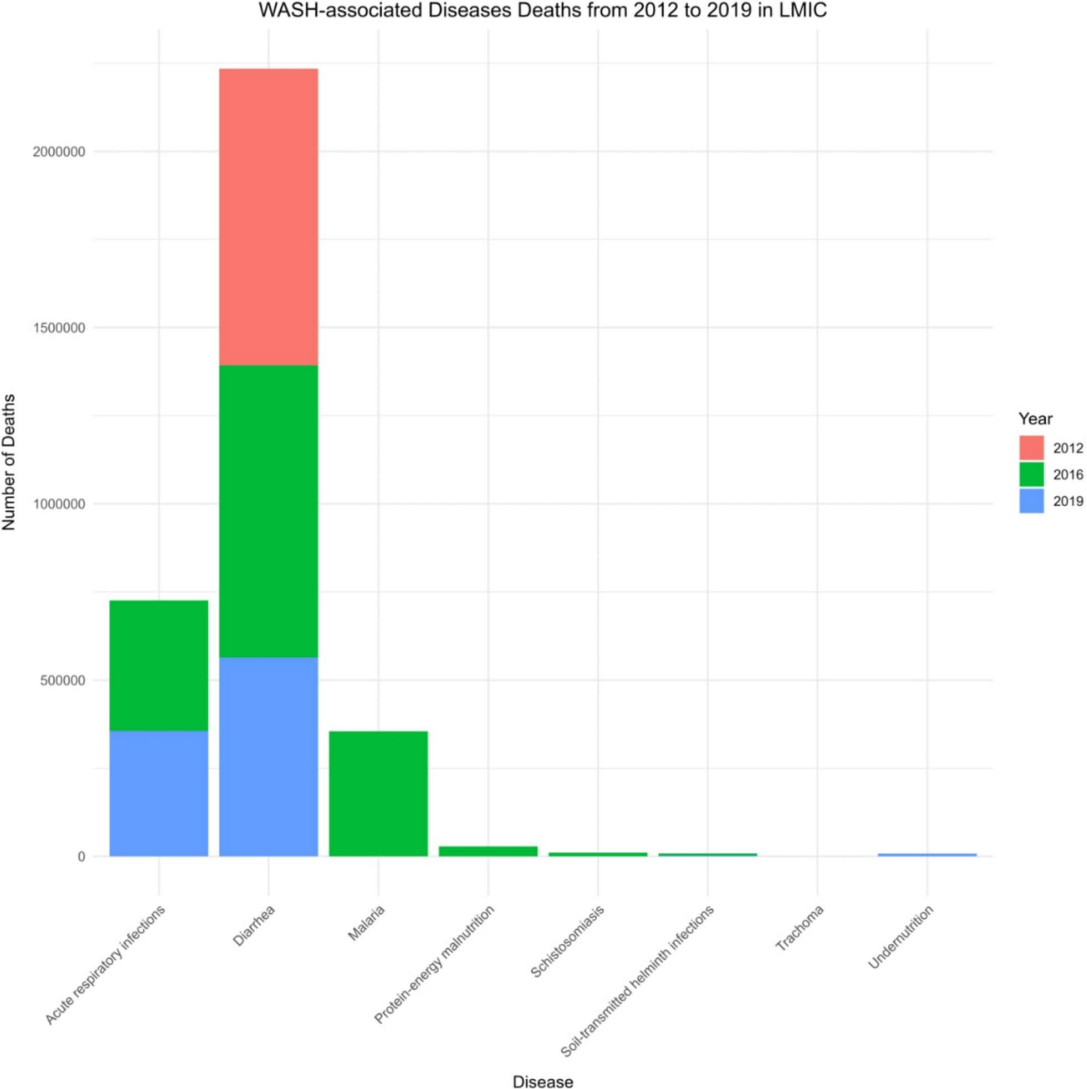
WASH-associated diseases from 2012 to 2019 in LMICs [9–11]

Similarly, WHO 2019 data reported a significant WASH-related disease burden in Africa; however, there were considerable reductions from earlier reporting periods. The continent experienced an alarming 1,035,000 deaths and 54,590,000 DALYs, with over 273,000 deaths among under-five children. Also, acute respiratory infections from poor hand hygiene resulted in 356,000 deaths, 16,578,000 DALYs, and 112,000 deaths among children under five. Undernutrition contributed remarkably to the burden, with 8,000 deaths and 825,000 DALYs, while soil-transmitted helminthiasis caused 2,000 deaths and 1,942,000 DALYs [11, 12] (Fig. 1).

## Challenges and barriers to the current landscape

Poverty, starvation, malnutrition, limited access to education, societal injustice, and isolation from decision-making all pose major barriers to WASH implementation in Africa. The "urbanization of poverty" contributes to slow sanitation improvement as heavily populated places strain sanitation systems. Poverty restricts communities' ability to practice proper hand hygiene, even when soap is present, due to limitations in accessing clean water and related sanitary tools [13]. Urban areas suffer from limited service availability, financial constraints, and institutional impediments prohibiting underprivileged populations from proper access. These problems emphasize the tangled relationship between poverty and WASH implementation in Africa [14]. Failures in water supply and sanitation systems, sustainability and maintenance of available WASH infrastructures, and socioeconomic and urbanization-based disparities in access are all obstacles to WASH policy in Africa [15]. Misgovernance, inadequate administrative institutions, and the need for stakeholder collaboration make policy implementation difficult [16]. Another major challenge is poor government involvement via appropriate ministries, as most hygiene efforts are spearheaded by non-governmental organizations (NGOs). This oversight impedes holistic efforts to provide safe water and enhance sanitation. Moreover, very few government efforts, such as that in Malawi—Malawi’s Hand Washing With Soap—are seen across the region [13]. This non-involvement limits effective planning, dedication, and budget allocation to improve hygiene behaviors and ensure long-term impact [13].

Implementing WASH policy in Africa and resultant monitoring and evaluation activities face stark setbacks, often compounded by inadequate data availability. Currently, less than half of UN member states have access to data on SDG 6 targets, and reliable data are essential for informed policymaking and investment in health. National challenges affect rural water system organization and policy integration. Robust data evaluation frameworks require a political commitment to transparency and the incorporation of earth observations, citizen science, and private sector data [17, 18]. Climate change presents substantial problems for Africa's WASH strategy, where sanitation access is inadequate. Nigeria, for example, has poorly managed systems and extensive rural settlements with insufficient access to water and sanitation, making it vulnerable to extreme natural conditions like flooding [19]. Changes in these conditions can impact WASH factors such as water treatment, sources, sanitation, hand hygiene, and storage, making it critical to implement effective and long-term strategies [20]. Inadequate funding undermines WASH initiatives, compromising water quality, sanitation, and hygiene practices, particularly in rural regions. Limited budgets contribute to inadequate infrastructure maintenance, resulting in damaged systems and contamination hazards [21, 22]. Population growth pressures urban WASH services, making providing clean water and maintaining hygiene standards difficult. Additionally, rapid urbanization and overcrowding exacerbate sanitation issues, emphasizing the importance of appropriate policies and infrastructure development to sustainably satisfy rising demand [21, 22]. Cultural beliefs and limited understanding of improved sanitation and hygiene hinder the adoption of healthy WASH practices, as some individuals and cultures are resistant to changing their ways [23–25]. Resistance to efficient WASH practices is common in areas where open defecation is robustly practiced. Geographical disparities in Africa, particularly in rural regions, pose significant challenges to implementing WASH regulations. This discrepancy impedes access to clean water and good sanitation, aggravating public health issues [2]. Cultural norms are often the root cause of resistance to new infrastructure, with varying viewpoints arising from differences in gender, ethnicity, and urban/rural divides [13]. Attitudes towards hygienic habits differ, making implementation problematic. Rural communities, for example, have switched from communal to individualized hand-washing habits in response to increased knowledge of communicable diseases such as cholera [2, 13].

## Implications to public health

Poor WASH practices in Africa lead to significant consequences, including health and socioeconomic disparities and various public health and environmental issues. According to WaterAid at the World Health Assembly 2024, over 2.2 billion people worldwide lack access to clean water [26]. This becomes worrisome as avoiding illnesses becomes extremely difficult, while compounding the rising burden of antimicrobial resistance (AMR), especially in Africa [26]. Inadequate WASH conditions make it harder for children, the elderly, pregnant women, and immunocompromised individuals to maintain good hygiene, increasing their vulnerability to preventable diseases caused by waterborne infections such as typhoid, dysentery, diarrhea, and neglected tropical illnesses such as trachoma, soil-transmitted helminths, and schistosomiasis. Inadequate WASH practices increase the risk of nosocomial infection and disease transmission, leading to elevated morbidity and mortality rates and further constraining existing healthcare settings [27]. The impact of inadequate WASH facilities on children is devastating, as over 700 children under the age of five succumb to fatal diarrheal infections every day in Africa. The implications are particularly severe in conflict zones, where children are approximately 20 times more likely to die from diarrheal infections than from the fighting itself [28, 29]. Teenage girls, especially those menstruating, face additional hurdles due to inadequate menstrual hygiene management facilities, which can impact their education, growth, and overall well-being [30]. The socioeconomically deprived, including refugees and asylum seekers, are disproportionately affected by poor WASH practices, as limited access to clean water and sanitation perpetuates poverty cycles and exacerbates existing inequalities [31]. The number of people at risk of water stress is projected to reach 5 billion by 2050 [20]. This growing threat is driven by excessive water extraction to meet population demands, which is exacerbated by consumption patterns, socioeconomic expansion, and climate change [20, 32]. Poorly managed human excreta causes environmental hazards, polluting settlements, groundwater, and surface water bodies [33]. Pollution negatively impacts ecosystems, biodiversity, and fisheries. This outcome is heightened in some regions and is promulgated by various factors, including wastewater management, climate, and chemical contamination from natural sources like arsenic and fluoride and man-made elements like nitrate [27]. Communities that depend on natural resources for their livelihoods face long-term dangers to sustainability and ecological balance due to environmental deterioration brought on by inappropriate waste disposal [34]. Heavily polluted coastal areas, such as those near large rivers, experience compromised fish catch, impacting local economies. The financial and economic consequences of poor WASH practices, including damage costs and broader welfare impacts, further underline the urgency for comprehensive interventions to address these complex issues, strengthen WASH practices and infrastructure throughout Africa, ensure the health and well-being of all population segments, and promote sustainable development on the continent [33, 35].

## The public health role of WASH

WASH plays a pivotal role in safeguarding health in Africa and lowering maternal and child morbidity and mortality globally. Preventing healthcare-associated infections (HAI) and combating infectious diseases like Ebola, COVID-19, cholera, and neglected tropical diseases could save lives and provide huge productivity gains. The absence of safe water, functional toilets, and proper handwashing facilities in healthcare settings poses significant risks to patients, healthcare workers, and communities [36]. Enhancing optimal WASH practices by patients and their healthcare providers, implementing stewardship programs, and improving environmental hygiene in health centers encompass strategies for breaking the chain of infection, and decreasing the spread of drug-resistant bacteria. Adequate infection prevention practices (IPC) reduced the need for medicines and indirectly reduced the possibility of antimicrobial resistance (AMR) by 85% [26]. Beyond healthcare, WASH contributes to overall public health by ensuring access to clean water, sanitation, and hygiene practices, fostering the attainment of the 2030 SDGs. Adequate water supply, sanitation systems, and effective infection prevention measures, including hand hygiene, are critical to reducing disease transmission. Furthermore, WASH initiatives play a role in improving the lives of female adolescents by addressing issues such as menstrual hygiene management, which is often overlooked but affects women's health and educational opportunities [37, 38]. Proximity to adequate WASH services, particularly piped water, is critical for societal welfare, which improves utilization, reducing the load on women and girls, who are mostly responsible for water collection in African settings. Improved sanitary infrastructures near residences provide privacy, comfort, and convenience, especially for vulnerable groups [39]. In educational settings, access to water, sanitation, and hygiene improves school attendance and performance, particularly for young girls, emphasizing the link between WASH and education [40]. While obstacles remain, understanding the complex effects of WASH initiatives is critical for promoting health, well-being, and gender equality in African communities.

**Fig. 2:**
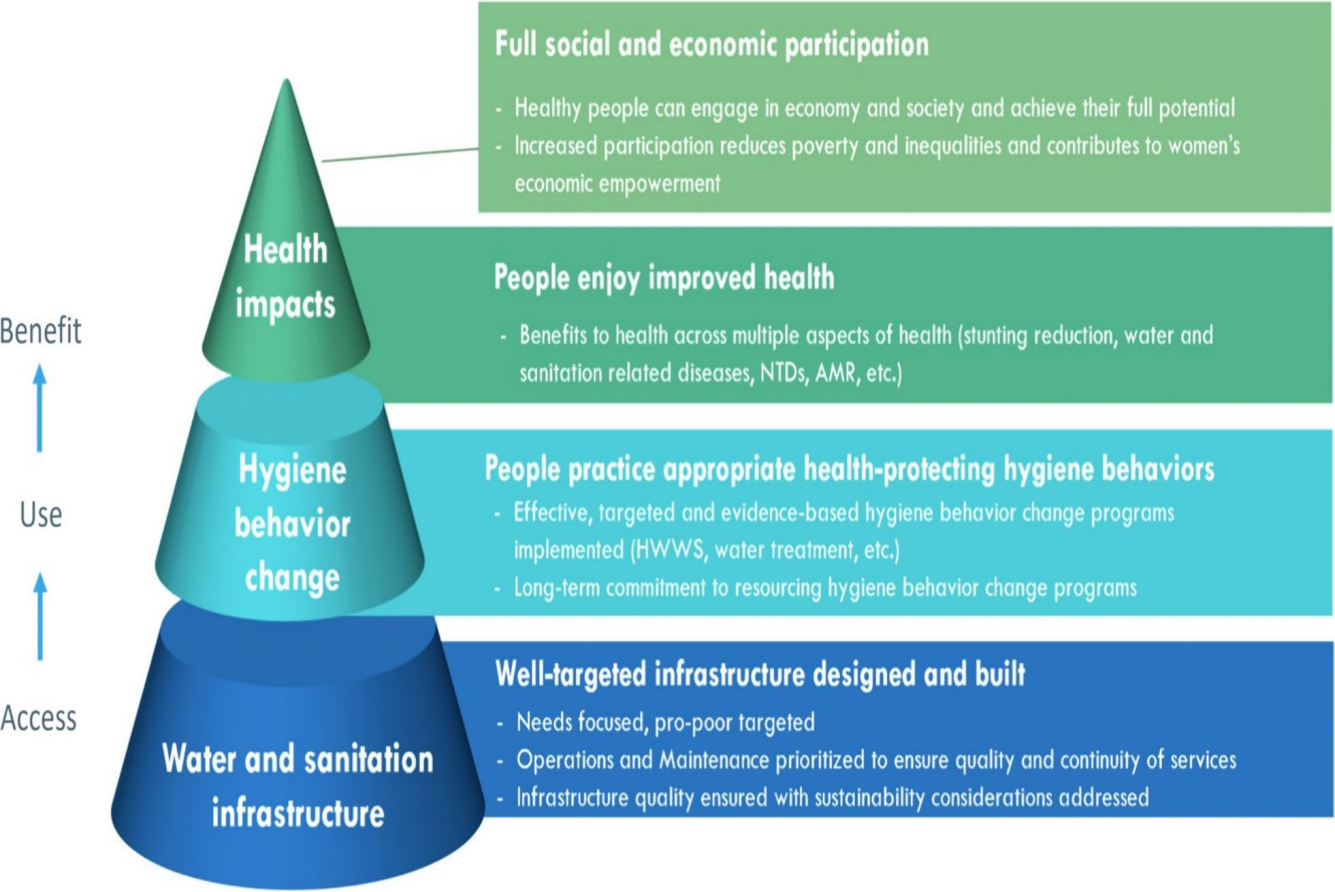
Basic role of WASH (extracted from 43)

Investing in water, sanitation, and hygiene (WASH) in Africa goes beyond satisfying basic needs; it emerges as a critical driver of public health, education, and economic growth. With potential financial returns of up to 700%, a US $1 investment in climate-resilient water and sanitation generates at least $7 [33]. Adequate WASH investments might help sub-Saharan Africa reap benefits equivalent to more than 5% of its GDP, or over $200 billion annually, thus accelerating economic growth. The benefits extend to gender equality since increased access empowers women and girls, lowering the 16 million hours spent daily fetching water and increasing education and employment prospects. In the face of climate change, population increase, and urbanization, WASH becomes critical to food security, SDG attainment, and sector-specific advances in agriculture, services, manufacturing, and energy [41, 42] (Fig. 2).

## Recommendation for public health: WASH approach

Africa's WASH approach should prioritize strong financial structures, recognizing rural WASH costs and equity in money allocation. Ministries should ensure resource allocation to enhance sanitation and hygiene practices and improve openness in the distribution of government funds to drive health processes. Equity in sector funding and careful consideration of various funding sources are critical for achieving national WASH goals, especially in rural communities [43]. Balancing subsidy approaches is critical for the scalability and long-term effectiveness of WASH efforts in Africa [44]. Addressing capacity deficiencies, particularly in training and facilitation skills, across the governmental and private sectors, civil society organizations (CSOs), and communities is necessary to improve WASH programs [45]. Community members and healthcare workers should participate in routine capacity-building training to increase community engagement, participation, and effective conduct of WASH activities [46]. The success of the 2020 Indonesia trial sets the path for the escalation of WASH FIT (Water, Sanitation, and Hygiene Facility Improvement Tool) to healthcare facilities (HCFs) in the country and other regions with substandard WASH activities [47]. In the course of this, ensuring an effective team constitution and adequate monitoring and evaluation activities for sustainability assures the feasibility of long-term goals [48]. Additionally, WaterAid emphasizes the importance of governmental administration, institutional capacity improvement, and additional financial resources. These are critical to resolving issues such as population growth, economic downturns, climate change, and rural-to-urban migration. They further recommend declaring WASH a top federation priority, setting explicit mandates, increasing funding, encouraging gender-responsive approaches, incorporating WASH into health policy, and focusing on climate resilience [49, 50].

Clear regulatory environments at regional, sub-national, and federal levels are needed to establish a strong framework beyond adopting agencies. A solid administrative and technical management framework with defined roles and duties at all levels of government is also needed to achieve effective service delivery [51]. Decentralizing WASH efforts can empower communities to take the initiative to enact laid-out guidelines. This strategy fosters self-reliance as well as addresses the obstacles experienced while redirecting and disseminating WASH services [52]. WASH implementation in Africa requires a complete plan that includes community ownership and active water management committees. The rural community's perspective is critical, emphasizing investing in water and sanitation facilities for long-term development [53]. A strong program methodology is vital for overcoming measurement issues and establishing best practices using qualitative methods such as “Most Significant Change (MSC) Stories” [54]. Effective indicators and evaluation methodologies are also required to replicate successful practices in rural water and sanitation programs [55, 56]. The success of WASH in Africa primarily relies on technology, such as community-managed water filters and hand-washing stations, gravity-fed systems, and latrines. These tools help people change their behavior and develop lasting habits. However, it is critical to address both initial adoption and long-term behaviors following the project. End-user-designed infrastructures are more suitable and sustainable. When technology is integrated with environmental conditions, its effectiveness increases, ensuring the long-term viability of WASH infrastructure [44, 56].

## Conclusion

Inadequate WASH practices have far-reaching consequences across Africa, exacerbating health disparities and financial obstacles while simultaneously sparking a range of environmental and public health issues. This condition increases susceptibility to waterborne illnesses, which mostly impact children, the elderly, pregnant women, and immunosuppressed people, exacerbating death rates and prolonging sickness cycles, especially in conflict zones. Teenage girls' well-being is further hampered by inadequate facilities for menstruation hygiene, while the socioeconomically disadvantaged are disproportionately affected. Water overuse and waste mismanagement threaten the ecological balance, the economy, and sustainability. In addition to preventing infections, optimal WASH is critical for effectively combating AMR. The overall effect highlights the necessity of all-encompassing interventions to strengthen WASH procedures, infrastructure, and sustainable development initiatives, guaranteeing the health and welfare of all African population segments.

## New findings of this paper

This study quantifies the morbidity and mortality burden associated with WASH-related diseases in LMICs, revealing a staggering amount of deaths and DALYs attributable to suboptimal WASH services. Temporal trends in WASH-related diseases are identified, indicating evolving challenges over time. The paper systematically identifies and discusses the barriers to WASH implementation, including poverty, urbanization, governance issues, and climate change impacts.

Furthermore, it assesses the wide-ranging public health implications of poor WASH practices, emphasizing their impact on disease transmission, environmental pollution, and economic well-being. Frequent optimal WASH practices by patients and their healthcare providers can all assist in breaking the chain of infection and pandemic preparedness, decrease the spread of drug-resistant bacteria, and reduce AMR by 85%.

## Data Availability

Not applicable.
